# Impostor phenomenon and psychological outcomes among family medicine residents: a cross-sectional study in Croatia

**DOI:** 10.2478/aiht-2025-76-3934

**Published:** 2025-06-30

**Authors:** Sunčana Vlah Tomičević, Valerija Bralić Lang

**Affiliations:** Health Centre Zagreb – East, Zagreb, Croatia; Private Family Physician Office, Zagreb, Croatia; University of Zagreb School of Medicine, Andrija Štampar School of Public Health, Department of Family Medicine, Zagreb, Croatia

**Keywords:** anxiety, depression, fraud, mental health, stress, anksioznost, depresija, mentalno zdravlje, stres, varalica

## Abstract

The aim of this cross-sectional study was to determine the prevalence of the impostor phenomenon among family medicine residents (FMRs) and its connection with sociodemographic factors and clinically relevant symptoms of depression, anxiety, and stress. During July 2023, 158 first-year FMRs were invited to fill out an anonymous online questionnaire containing sociodemographic data, history of psychiatric disorders, Clance Impostor Phenomenon Scale (CIPS), and the 21-item Depression Anxiety Stress Scale (DASS-21). Seventynine participants responded (50 % response rate) and 59 reported some level of impostor feelings. Clinically relevant symptoms of depression were reported by 17, anxiety by 23, and stress by 20 participants. All 59 participants whose responses indicated impostor feelings above normal also had higher scores for depression, anxiety, and stress (p<0.001 in Mann-Whitney *U* test). Despite a small sample with uneven gender distribution and self-reported scales, our study found a significant association between the impostor phenomenon and negative mental outcomes. We believe that the impostor phenomenon among FMRs can be addressed effectively with interventions like peer support, mentoring, and practicing self-compassion if started early during medical study and specialisation.

## Abstract (hr)

Cilj ovoga presječnog istraživanja bio je utvrditi prevalenciju fenomena varalice (eng. *impostor phenomenon*) među specijalizantima obiteljske medicine i istražiti povezanost sociodemografskih čimbenika i klinički značajnih simptoma depresije, anksioznosti i stresa. Tijekom lipnja 2023. godine 158 specijalizanata obiteljske medicine ispunili su anonimni elektronički anketni upitnik koji je sadržavao pitanja o sociodemografskim podacima, povijesti psihijatrijskih poremećaja, Clanceovu ljestvicu fenomena varalice (CIPS) te DASS-21 ljestvicu o depresiji, anksioznosti i stresu. Sudjelovalo je 79 ispitanika (50 % stopa odaziva), a 59 je imalo određenu razinu osjećaja da su varalice. Klinički značajne simptome depresije imalo je 17, anksioznosti 23, a stresa 20 ispitanika. Svih 59 ispitanika koji su imali osjećaj da su varalice, imali su i viši rezultat za simptome depresije, anksioznosti i stresa (p<0,001 prema Mann-Whitney *U* testu). Iako se radilo o malom uzorku, većinom žena, nađena je povezanost između osjećaja varalice i negativnih psiholoških ishoda. Intervencije poput kolegijalne potpore, mentorstva i vježbanja samosuosjećanja tijekom školovanja omogućile bi ublažavanje fenomena varalice.

Mental health difficulties are common among doctors, especially women and young doctors (1). Some conditions like impostor phenomenon or syndrome (IP) can affect mental health, and physicians seem to be more susceptible to it, especially during residency (2,3,4). IP was first described by Clance and Imes (5) in 1978 as a feeling that one’s own success is owed to luck or a mistake or other reason rather than one’s own merit. Hence the feeling of being a fraud. IP is not considered a mental illness but a set of personality characteristics that can heighten during schooling and lead to over-preparedness or procrastination by working overtime to meet the last-minute deadline (4, 6). It starts at a young age, increases during schooling, especially if problems or difficulties are not addressed properly, and is considered prominent among young people, highly successful individuals, women, and minority groups (7, 8).

In Croatia, family medicine residents (FMRs) often work as the sole responsible physicians in family medicine for several years, even before beginning their residency. Since they start working in general practice immediately after completing medical school, they lack necessary education and skills to function as independent family medicine doctors. Administrative burden and high patient load they face pose a risk of poor mental outcomes as they exact high mental strain and a feeling of displacement, which favours the occurrence of IP (9, 10). Since young doctors have a higher prevalence of burnout, anxiety, and depression, a pre-existing condition like IP should be considered as a possible contributor to these negative mental states (11).

The aim of our cross-sectional study was to test this hypothesis by determining the prevalence of IP among first-year FMRs and its association with clinically relevant symptoms of depression, anxiety, and stress.

## PARTICIPANTS AND METHODS

For this anonymous survey we invited 158 first-year FMRs in Zagreb, Croatia in July 2023. Only the first-year FMRs were included, because IP is more prominent in younger doctors, and they all attended the theoretical part of the specialisation and were available for this study (2).

The participants were recruited via email with a link to the web version of the questionnaire. All the invited participants were instructed to fill the survey only once. After three reminders, we closed the survey, having received completed questionnaire from 79 respondents (50 % response rate).

The survey was approved by the University of Zagreb School of Medicine Ethics Committee (approval No. 251-59-10106-24-111/150 of 9 October 2023).

### Questionnaire

The questionnaire we developed specifically for this study is in Croatian and designed to collect sociodemographic data (age, gender, years of work, partner status, having children or not), history of psychiatric disorders, and whether the study of medicine and specialisation in family medicine were the participants’ first career choice.

Additionally, the questionnaire incorporated two instruments: the 20-item Clance Impostor Phenomenon Scale (CIPS) and the 21-item Depression Anxiety Stress Scale (DASS-21). We used the Croatian version of CIPS (12) to determine the level of IP in respondents as described by Clance (13). Each item is answered on the Likert scale from 1 (not true at all) to 5 (very true) and each score added. If the total score is 40 or less, the respondent seldom has impostor feelings; a score between 41 and 60 corresponds to a moderate frequency of IP experience; a score between 61 and 80 indicates that the respondent often has impostor feelings; and a score higher than 80 means the respondent often has intense impostor feelings. DASS-21 quantifies symptoms of depression (severity cut-off score >9), anxiety (cut-off score >7), and stress (cut-off score >14) (14, 15).

### Statistical analysis

Before we started analysing data, we ran a power analysis with an independent sample *t*-test. Our sample of 79 participants is above the requirement of 70 for the minimum desired power of 0.9, moderate effect size of 0.5, and α of 0.05.

We analysed the obtained data using the jamovi^®^ version 2.6 computer software (open source). As the Kolmogorov-Smirnov test showed that data distribution was not normal (tested for each variable), we used the Mann-Whitney *U* test to compare two groups and the Kruskal-Wallis test to compare three or more groups. Quantitative data are presented as medians and ranges (interquartile where appropriate). The p-value of <0.05 was considered significant. To see how independent predictors like gender, partner status, having children, and having a history of psychiatric disorders affected DASS-21 and CIPS scores, we ran univariate logistic regression.

## RESULTS

There were 65 women in the sample. The median age for both genders is 30, ranging from 26 to 50 years for women and from 27 to 43 years for men. Forty-nine respondents had worked for less than five years. Seventy-four claimed the study of medicine was their first choice and 72 picked family medicine as their first choice for specialisation. Forty-one had a partner, and 22 had at least one child. Six participants reported a history of psychiatric disorders (Table 1).

**Table 1:** Selected characteristics of study participants (N=79)

**Variable**	**Male** **N**	**Female** **N**
Partner		
Yes	5	36
No	9	29

Children		
Yes	1	21
No	13	44

Mental illness		
Yes	2	4
No	12	61

School of medicine as first choice		
Yes	14	60
No	0	5

Family medicine residency as first choice		
Yes	13	59
No	1	6

Table 2 shows the distribution of participants of both genders by the frequency and severity of experienced impostor feelings. Moderate to intense IP (CIPS>40) was reported by 59 participants (Cronbach’s alpha 0.95).

**Table 2:** Prevalence of impostor feelings among Croatian family medicine residents (N=79)

**CIPS (Impostor feelings)**	**N**	**Median score (min–max)**	**95 % CI**
Few ≤40	20	31.5 (21–40)	28.1–33.3
Moderate 41–60	33	48 (41–60)	46.7–50.5
Frequent 61–80	19	66 (62–80)	66.0–72.0
Intense >80	7	89 (81–99)	81.9–93.8

Clance Impostor Phenomenon Scale (CIPS) score categories: few impostor characteristics ≤40; moderate impostor experiences 41–60; frequent impostor feelings 61–80; intense impostor experiences >80. CI – confidence interval

Mild to extremely severe symptoms of depression were reported by 17 participants, anxiety by 23, and stress by 20 participants (Cronbach’s alpha 0.91 for depression, 0.79 for anxiety, and 0. 87 for stress) (Table 3).

**Table 3:** Prevalence of depression, anxiety, and stress by severity among Croatian family medicine residents (N=79)

**DASS-21 scores**	**Normal (N)**	**Mild (N)**	**Moderate (N)**	**Severe (N)**	**Extremely severe (N)**	**Median score (min–max)**
Depression	62	5	9	1	2	2 (0–34)
Anxiety	56	5	14	2	2	4 (0–24)
Stress	59	6	12	1	1	10 (0–38)

DASS-21 cut-off scores for 1) depression: ≤9 normal; 10–13 mild; 14–20 moderate; 21–27 severe; 28–42 extremely severe; 2) anxiety: ≤7 normal; 8–9 mild; 10–14 moderate; 15–19 severe; 20–42 extremely severe; and 3) stress: ≤14 normal; 15–18 mild; 19–25 moderate; 26–33 severe; 34–42 extremely severe

There were no significant differences in CIPS, depression, anxiety, or stress scores between men and women (p=0.059, p=0.905, p=0.228, and p=0.792, respectively). With the median CIPS score of 44 (IQR 43.1–50.4) participants with a partner also did not significantly differ from those without a partner (p=0.052). The same is true for participants who had children (p=0.053). History of psychiatric disorders also did not affect CIPS, depression, anxiety, or stress scores (p=0.556, p=0.200, p=0.962, p=0.340, respectively) (Table 4). The same is true for participants whose first choice was the study of medicine and specialisation in family medicine (p=0.724, p=0.724, p=0.246, and p=0.571, respectively).

**Figure 1:**
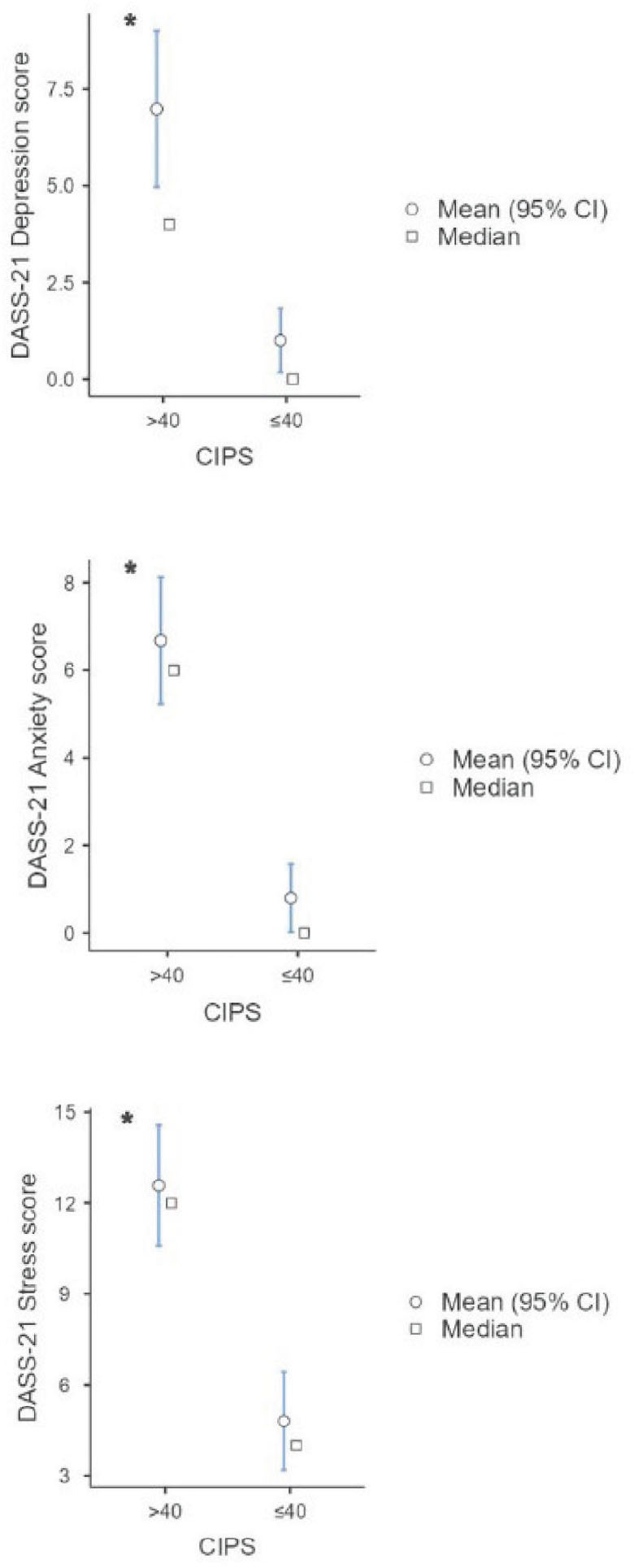
Plots showing higher mean (95 % CI) and median values of DASS-21 depression, anxiety, and stress scores in participants with CIPS scores higher than 40. * p<0.001 (Mann-Whitney *U* test). CI – confidence interval; CIPS – Clance Impostor Phenomenon Scale; DASS-21 – Depression Anxiety Stress Scale 21

Participants with higher CIPS scores also scored higher on all three subscales of DASS-21 (p<0.001) (Figure 1).

**Table 4:** Correlation between sociodemographic variables and CIPS and DASS-21 scores

	**OR_uv_**	**95 % CI**	p-value^*^
**CIPS**			
Gender			
Male/Female	0.54	0.16–1.86	0.329
Partner			
Yes/No	0.36	0.12–1.07	0.066
Children			
Yes/No	0.34	0.12–1.01	0.053
Mental illness			
Yes/No	1.76	0.19–16.03	0.616
**DASS-21 Depression**			
Gender			
Male/Female	0.99	0.24–4.05	0.993
Partner			
Yes/No	0.42	0.14–1.28	0.128
Children			
Yes/No	0.75	0.22–2.62	0.655
Mental illness			
Yes/No	4.21	0.77–23.14	0.098
**DASS-21 Anxiety**			
Gender			
Male/Female	0.35	0.07–1.70	0.193
Partner			
Yes/No	1.02	0.38–2.68	0.975
Children			
Yes/No	0.88	0.29–2.64	0.823
Mental illness			
Yes/No	1.24	0.21–7.28	0.813
**DASS-21 Stress**			
Gender			
Male/Female	1.22	0.34–4.45	0.758
Partner			
Yes/No	0.89	0.25–1.91	0.476
Children			
Yes/No	0.57	0.17–1.94	0.369
Mental illness			
Yes/No	0.57	0.06–5.18	0.616

* – univariate logistic regression.

CI – confidence interval; CIPS – Clance Impostor Phenomenon Scale; DASS-21 – Depression Anxiety Stress Scale 21; OR_uv_ – odds ratio obtained with univariate logistic regression

## DISCUSSION

This cross-sectional study in a sample of 79 first-year FMRs shows that most participants had some level of impostor feelings and that all participants with frequent or intense impostor feelings (more than one third) had significantly higher DASS-21 scores, which suggests that the impostor phenomenon increases the risk of stress, anxiety, and depression.

Our finding that more than one-third of participants had frequent or intense impostor feeling is consistent with the reported prevalence in medical students, residents, and physicians, ranging from 22.5 % to 46.6 % in different studies (16,17,18).

In contrast, DASS-21 scores in our sample are lower than the worldwide prevalence among resident physicians (19) but are still higher than in the general population (20). Some studies argue that the cause for poor psychological outcomes and impostor feelings is not working in a specific medical field but workplace-related risk factors like job demands, work-job imbalance, working overtime, administrative work, and, in the case of medical residents, inadequate training and the pressure that comes with studying and exams (8, 18). However, family medicine doctors in Croatia fit the bill: they often work overtime, they are burdened with administrative work, and receive a large number of patients in everyday practice. The reason for lower prevalence of depression, anxiety, and stress symptoms in our study sample could be that most of them had worked for less than five years in their profession. Also, they were surveyed at the beginning of their residency, when the workload and study/exam stress had not reached the peak.

The impostor syndrome had long been more common in women, but recent research confirms our findings, however limited, that this is no longer the fact (7, 18). The reason could be a shift in the gender paradigm, as more and more women work as physicians, breaking thus the stereotype of women as dominantly nurses (7). In fact, it is more common for a family doctor in Croatia to be a woman (21, 22), which is perhaps why our women FMRs do not feel like impostors based on their gender.

Other factors were not significantly associated with the CIPS scores, which is supported by various studies that found years of work, peer support, regular feedback, and good mentorship to have more impact on the result (11, 23).

The key finding of our research – the significant association between higher CIPS and DASS-21 scores – however, has confirmed our assumption that impostor feelings are associated with depression, anxiety, and stress. It also confirms earlier reports of IP being linked with increased burnout, anxiety, depression, suicide, and lower professional fulfilment (23, 24). More impostor feelings mean more psychological distress, as pointed out in a study among medical students (25) and among nursing students in Egypt (26). Another study on university students in the Netherlands suggests that higher levels of IP lead to mental exhaustion, anxiety, and fear of failure (27).

Considering that the impostor feeling is often heightened during medical schooling, addressing this issue should start at schools (28). Most studies show the impostor feelings can be alleviated with positive feedback and affirmation, safe environment to discuss medical errors, preparation for patient complaints, institutional support, and by avoiding delayed gratification (4, 7, 29). Changes should be made on institutional and personal levels to improve doctors’ mental health, with the emphasis on good family relationships and work-life balance as an essential part of medical school and residency curriculum.

### Limitations of the study

The first limitation of this study is its cross-sectional design, which does not allow conclusions about causality. This is also the first study of this kind in FMRs in Croatia, and no comparison with earlier findings is possible. The third limitation is that both CIPS and DASS-21 rely on self-reported data and may be prone to response bias (withholding information on the history of psychiatric issues) or selection bias, considering that the response rate was 50 % (suggesting that only intrinsically motivated participants responded). Finally, the major limitation of our study is the possible sample bias owed to the small sample and the uneven distribution of participants by gender. It may have affected the findings of associations between specific parameters (such as gender, history of psychiatric issues, parenting, or having a partner) and the CIPS and DASS-21 scores. However, we find this sample representative of FMRs in Croatia, as generally 30–40 medical doctors enrol for this specialisation per year, and they are mostly women.

## CONCLUSION

Despite the above limitations, our study has confirmed the association between impostor feelings and depression, anxiety, and stress in Croatian first-year FMRs. To our knowledge, this is the first study conducted among FMRs in Croatia to examine the association between the impostor phenomenon and mental health outcomes. Future research should aim to further investigate the prevalence, predictors, and consequences of the impostor phenomenon in this population, as well as its impact on residents’ well-being, clinical performance, and professional development. Strategies to alleviate IP among FMRs may include encouraging help-seeking, constructive feedback, recognising achievements, fostering peer support, promoting cognitive approaches such as reframing negative thoughts, practicing self-compassion, and implementing interventions such as small group discussions, workshops, and mentoring programmes. Follow up on the effectiveness of such strategies and interventions should also be part of future research to identify best practices in reducing impostor feelings.
